# Biosynthesis and Functions of Very-Long-Chain Fatty Acids in the Responses of Plants to Abiotic and Biotic Stresses

**DOI:** 10.3390/cells10061284

**Published:** 2021-05-21

**Authors:** Marguerite Batsale, Delphine Bahammou, Laetitia Fouillen, Sébastien Mongrand, Jérôme Joubès, Frédéric Domergue

**Affiliations:** University of Bordeaux, CNRS, Laboratoire de Biogenèse Membranaire, UMR 5200, F-33140 Villenave d’Ornon, France; marguerite.batsale@u-bordeaux.fr (M.B.); delphine.bahammou@u-bordeaux.fr (D.B.); laetitia.fouillen@u-bordeaux.fr (L.F.); sebastien.mongrand@u-bordeaux.fr (S.M.); jerome.joubes@u-bordeaux.fr (J.J.)

**Keywords:** very-long-chain fatty acids, surface lipids, sphingolipids, elongation complex, stress response, *Arabidopsis*

## Abstract

Very-long-chain fatty acids (i.e., fatty acids with more than 18 carbon atoms; VLCFA) are important molecules that play crucial physiological and structural roles in plants. VLCFA are specifically present in several membrane lipids and essential for membrane homeostasis. Their specific accumulation in the sphingolipids of the plasma membrane outer leaflet is of primordial importance for its correct functioning in intercellular communication. VLCFA are found in phospholipids, notably in phosphatidylserine and phosphatidylethanolamine, where they could play a role in membrane domain organization and interleaflet coupling. In epidermal cells, VLCFA are precursors of the cuticular waxes of the plant cuticle, which are of primary importance for many interactions of the plant with its surrounding environment. VLCFA are also major components of the root suberin barrier, which has been shown to be fundamental for nutrient homeostasis and plant adaptation to adverse conditions. Finally, some plants store VLCFA in the triacylglycerols of their seeds so that they later play a pivotal role in seed germination. In this review, taking advantage of the many studies conducted using *Arabidopsis thaliana* as a model, we present our current knowledge on the biosynthesis and regulation of VLCFA in plants, and on the various functions that VLCFA and their derivatives play in the interactions of plants with their abiotic and biotic environment.

## 1. VLCFA Structure, Biosynthesis and Distribution

As sessile organisms, plants depend on physiological and structural mechanisms allowing them to adjust to their ever-changing environment. Some of those mechanisms involve essential lipid molecules throughout the course of plant development such as very-long chain fatty acids [1,2,3,4,5]. With their acyl chain of 20 carbons and longer, VLCFA can be modified and derivatized into very flexible components and precursors of various important lipids [6,7]. Indeed, they are required in all plant cells for the synthesis of essential membrane lipids such as phospholipids [8] and sphingolipids (mostly in plasma and vacuolar membranes) [9,10,11,12]. They are also used as precursors of protective cuticular waxes and suberin in leaves epidermis and roots, respectively [13,14,15,16,17]. Finally, they can also be incorporated in seeds triacylglycerols (TAGs) towards the accumulation of storage lipids [18,19]. Plant VLCFA are therefore precursors of abundant lipids involved in different physiological processes such as membrane trafficking, cell division and differentiation, non-stomatal water loss prevention, or energy storage [2,3,18,20]. Hence, VLCFA display structural, protective, nutritive and energetic properties that have already led to many reports on their synthesis and functions in plants.

### 1.1. VLCFA Definition and Chemical Diversity

**Figure 1:**
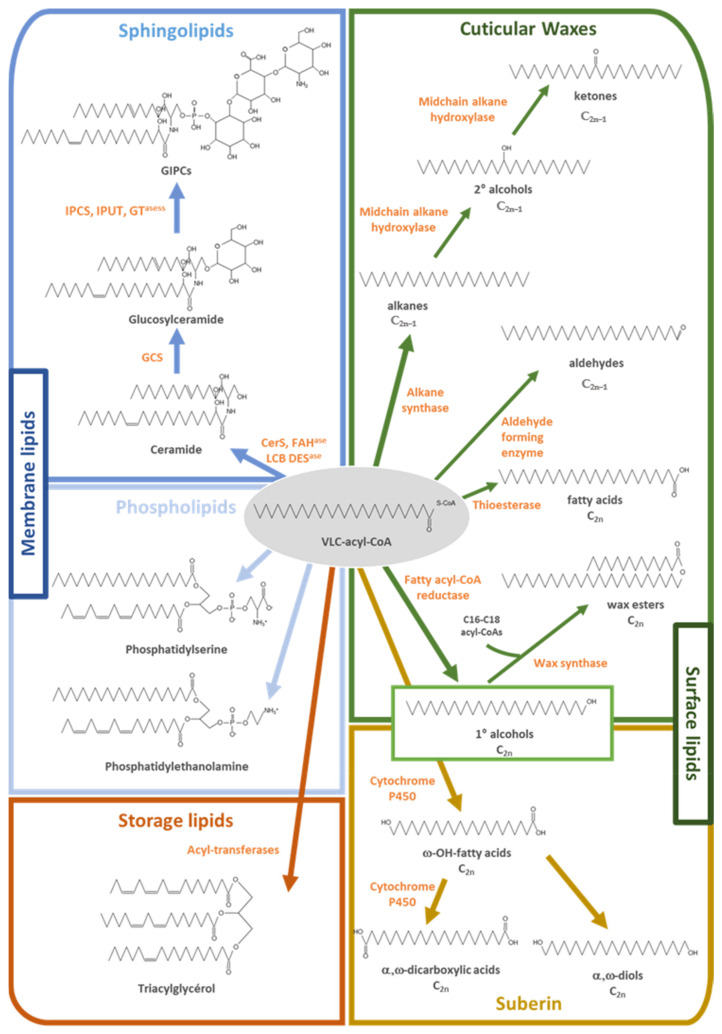
Metabolic fates of very long-chain acyl-CoAs. VLC-acyl-CoA produced by fatty acid elongation complexes can be converted into aliphatic derivatives incorporated into the cuticle as cuticular waxes or serve as building blocks for the suberin biopolyester biosynthesis. VLC-acyl-CoA can be incorporated into storage lipids as triacylglycerols or in membrane lipids such as phospholipids (phosphatidylserine and phosphatidylethanolamine) or sphingolipids (ceramide, glucosylceramide and GIPCs). Abbreviations: CerS, ceramide synthase; FAH^ase^, fatty acid hydroxylase; GCS, glucosylceramide synthase; GIPCs, glycosyl-inositolphosphoryl-ceramides; GT^ases^, glycosyl-transferases; IPCS, inositolphosphoryl-ceramide synthase; IPUT, inositolphosphoryl-ceramide glucuronosyl-transferase; LCB DES^ase^, LCB desaturase; VLC-acyl-CoA, very-long-chain-acyl-CoA.

Very-long-chain fatty acids are simply defined as fatty acids with more than 18 carbon atoms, but the lengths of the acyl-chain, its unsaturation degree and hydroxylation levels are at the origin of an extremely high diversity of VLCFA and derivatives in plants (Figure 1).

Membrane phospholipids such as PhosphatidylCholine (PC), PhosphatidylEthanolamine (PE) and especially PhosphatidylSerine (PS) preferentially incorporate saturated C20, C22 and C24 VLCFAs [8], while sphingolipids accumulating in the outer leaflet of the plasma membrane (PM) are often enriched in α-hydroxylated saturated and monounsaturated C24 and C26 VLCFAs [10]. Although most intracellular VLCFA have twenty-six or fewer carbon atoms (≤C26), plant cuticular VLCFA and derivatives generally have around 30 carbon atoms, while leaf trichomes and pavement cells have been shown to produce VLCFA with up to 38 carbon atoms [21]. If plant species display variable amounts of free VLCFA in their cuticular waxes, all aliphatic wax compounds are derived from VLCFA, and cuticular waxes usually comprise a wide variety of chain-length and chemical structures: aldehydes, primary and secondary alcohols, alkanes, ketones and wax compounds with two secondary functional groups in some plant species [22]. If most wax VLCFA derivatives are saturated, minor amounts of unsaturated alcohols and alkenes as well as branched molecules occur in *Arabidopsis* [21,23,24]. Therefore, although 10 to 20 VLCFA derivatives are usually reported as major aliphatic components of arabidopsis waxes, their complete composition is unambiguously much more complex. Like in sphingolipids, hydroxylated VLCFA have been reported to be abundant in suberin, but in this case they are ω-hydroxylated VLCFA. In addition, suberin is known to contain high amounts of VLCFA-derived α,ω-dicarboxylic acids, alcohols and diols [14,15,25]. Finally, VLCFA can also be accumulated in storage TriAcylGlycerols (TAG). In arabidopsis seed storage lipids, VCLFA represent about 27% of the total acyl-chains, and if C20 and C22 monounsaturated VLCFA dominate, polyunsaturated VLCFA with up to three double bonds are also present in minor amounts [19]. It should be remembered that each modification of the acyl-chain has direct impacts on the chemical and physical properties of the resulting fatty acid and derivatives, enabling a great diversity of lipid functional specializations [26].

### 1.2. VLCFA Biosynthesis

**Figure 2:**
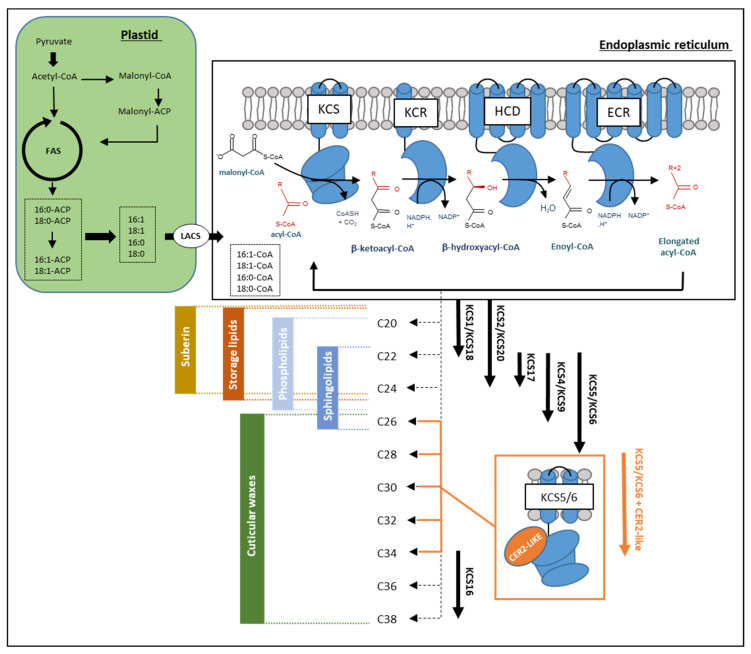
Biosynthesis and selective involvement of VLC acyl-CoAs in the different lipid biosynthesis pathways in *Arabidopsis*. VLC acyl-CoAs are elongated from C16 and C18 long-chain fatty acids (LCFAs) synthesized in the plastid by the fatty acid synthase (FAS) complex. Plastidial saturated and monounsaturated C16 and C18 fatty acids are exported to the cytosol, where they are activated as acyl-CoAs by Long-Chain Acyl-CoA Synthetases (LACs). C16- and C18-CoA are then elongated into very-long-chain acyl-CoAs by the fatty acid elongation (FAE) complex. This complex consists of four enzymes localized in the reticulum endoplasmic membrane. Four sequential reactions lead to the addition of two carbon units: a condensation, a reduction, a dehydration and a final reduction, respectively, catalyzed by a β-Keto-acyl-CoA Synthase (KCS), a β-Keto-acyl-CoA Reductase (KCR), a 3-Hydroxyacyl-CoA Deshydratase (HCD) and an Enoyl-CoA Reductase (ECR). Different FAE complexes with different AtKCSs coexist in a same cell to produce VLC acyl-CoAs from C20 to C38. The resulting pool of acyl-CoAs is exploited towards the synthesis of different lipid categories such as membrane lipids (sphingolipids and phospholipids), surface lipids (cuticular waxes and suberin) and storage lipids (TAG). Abbreviations: ACP, acyl-carrier protein; CoA, Coenzyme A.

Studies, including photoperiod and chemical inhibition of elongase activities, initially raised the idea for the existence of multiple fatty acid elongase complexes (FAE complex) that perform sequential and/or parallel reactions to produce the broad chain-length-range of VLCFAs found in plants [27]. VLCFA biosynthesis begins with the elongation of saturated and monounsaturated C16 and C18 fatty acids produced in the plastid and exported to the cytosol where they are activated as acyl-CoAs by long-chain acyl-CoA synthetases [28,29]. These long-chain acyl-CoAs represent the immediate precursors of VLCFA and are further elongated through FAE complexes localized in the endoplasmic reticulum membranes (Figure 2; [7]). Biochemically, four core enzymes perform in a FAE complex four sequential reactions to extend the acyl-chain by two carbon units. The first reaction consists of a condensation of the acyl-CoA substrate (n) with a malonyl-CoA catalyzed by a condensing enzyme called β-Keto-acyl-CoA Synthase (KCS) to give a β-Keto-acyl-CoA (n+2) [30]. A β-Keto-acyl-CoA Reductase (KCR) reduces the β-keto group into an alcohol, using NAD(P)H as a reductant, to produce a hydroxyacyl-CoA [31]. A 3-Hydroxyacyl-CoA Deshydratase (HCD) then converts this intermediate into an enoyl-CoA [32], which is reduced by an Enoyl-CoA Reductase (ECR) to form a saturated fatty acyl-CoA (n+2) product [33]. This reaction cycle can be repeated to yield VLCFA with various chain lengths ranging from C20 up to C38 or more. 

*KCS18*, initially called *FAE1*, was the very first FAE gene to be investigated and cloned in plants [34]. The demonstration of its involvement in the biosynthesis of the VLCFA present in arabidopsis seeds storage lipids represents a major step towards our understanding of VLCFA elongation process in plants. In the last two decades, major findings on VLCFA synthesis in yeast allowed for the identification of the genes coding for KCR, HCD and ECR enzymes [35,36,37], and subsequently their homologs in arabidopsis [31,32,33]. Complementation assays of yeast mutants revealed that *KCR1* and *PASTICCINO2 (PAS2)* encode functional KCR and HCD enzymes, respectively [31,32]. Complementation of the arabidopsis *eceriferum10* (*cer* meaning “not bearing waxes”, *cer10*) mutant and the yeast *tsc13-1 elo2Δ* mutant by expressing *AtECR* demonstrated that *CER10* encodes a functional ECR [33]. Phylogenetic analyses then emphasized the variable genetic diversity of the FAE components across different plant species. Whereas the ECR subunit is often encoded by a single-copy gene, the condensing enzyme KCS is usually encoded by a very large multi-copy gene family: 21 putative isoenzymes were identified in arabidopsis [30], 28 in maize (*Zea mays*) [38], 30 in peanut (*Arachis hypogea*) [39] and as much as 58 in rapeseed (*Brassica napus*) [40]. Multiple-copy genes can encode the KCR and HCD components, but with a much lower diversity. In maize, ZmKCR1 and ZmKCR2 are both able to catalyze the reduction of 3-ketoacyl-CoA intermediates with different chain-length outcomes [38,41]. However, only one duplicated homolog from arabidopsis shows this catalytic potential [31]. The HCD component can either be a single-copy gene as in tomato (*Solanum lycopersicum***)** and maize or multi-copy genes like in rice (*Oryza sativa japonica*; 6 genes) and sorghum (*Sorghum bicolor*; 3 genes; [38]). In arabidopsis, PASTICCINO2 (PAS2) was characterized as the major HCD isoenzyme [32], but the PROTEIN TYROSIN PHOSPHATASE-LIKE (PTPLA) was later suggested as the HCD of specific FAE complexes expressed in vascular tissues that regulate endodermal VLCFA elongation by a yet-to-be-discovered non-cell autonomous mechanism [42]. Overall, the rather low genetic diversity found in *KCR*, *HCD* and *ECR* genes suggests that these three subunits generate large ranges of VLCFA chain lengths through broad substrate specificities, while KCS enzymes determine the chain-length substrate specificity of each elongation reaction. Consistent with the assumption that KCR, HCD and ECR enzymes may be common to all FAE complexes, the total loss of KCR1 or PAS2 is embryo lethal, while the loss of CER10 or partial loss of KCR1 or PAS2 activity results in a severe reduction of all major classes of VLCFA-containing lipids (waxes, triacylglycerols and sphingolipids), causing major developmental impairments [31,32,33]. In contrast, none of the *kcs* mutant showed lethality in arabidopsis.

In addition to the four core enzymes forming FAE complex, other proteins were shown to play important functions, including CER2-LIKE proteins, a group of five proteins homologous to BAHD acyltransferases [43]. CER2 was first investigated in the mutant *eceriferum2* (*cer2*) deficient in specific VLCFA-derived waxes longer than C28. Despite being homologous to BAHD acyltransferases family (BAHD for BEAT, AHCT, HCBT, and DAT family of proteins), the conserved amino acids of the acyltransferase catalytic site are not required for CER2-LIKE proteins activity in cuticular wax biosynthesis [43,44]. The variable product specificity of CER2-LIKE proteins has been deciphered using yeast as heterologous system. Co-expression of CER2-LIKE proteins with KCS6 or KCS5 leads to different VLCFAs accumulation profiles. CER2-LIKE4 seems to be involved in the elongation of C26 into C28, CER2 and CER2-LIKE3 modulate KCS6 elongation specificity to produce C30 VLCFAs, while CER2-LIKE2 enables the elongation of VLCFAs up to C32 and CER2-LIKE1 up to C34 VLCFAs (Figure 2; [43,45]). How these CER2-LIKE proteins affect KCS chain length specificity remains to be elucidated. The arabidopsis PAS1 and AKR2A proteins, which are molecular chaperones, are other examples of FAE complex regulators that are known to target protein complexes and regulate their assembly or activity [2,46].

### 1.3. Arabidopsis KCS Multigenic Family

As stated above, 20 *KCS* genes are present in the genome of arabidopsis, and the exact biochemical activity and in planta function of the encoded proteins have been mainly studied through heterologous expression in yeast and characterization of Arabidopsis mutant lines.

Since the yeast VLCFA elongation system is very similar to plant FAE complex, several AtKCSs have been shown to be active in yeast wild-type and *Δelo3* backgrounds, and the chain length of the VLCFA they produce could be determined in vivo [47,48] or in vitro [49,50]. Functional characterization of AtKCSs chain-length specificity has been thoroughly investigated by Tresh and coworkers [48]. Nevertheless, although they expressed 17 out of 20 AtKCS in yeast, they could confirm the activity of only 7 of them: KCS1 and KCS18, which mainly produced C20 and C22 VLCFA; KCS2 and KCS20, mainly producing C22 and C24 VLCFA; KCS5 and KCS6, mainly producing C24 to C28 VLCFA; and KCS17, mainly producing C24 VLCFA (Figure 2). Another KCS from arabidopsis was more recently characterized in yeast as Hegebarth and coworkers [51] showed that AtKCS16 was able to produce VLCFA up to 38 carbons when co-expressed with KCS6 and CER2-LIKE1, which most probably provided the required C34 acyl-CoA substrate (Figure 2). Finally, in vitro assays using microsomes isolated from yeast expressing an arabidopsis *KCS* confirmed the chain length specificity of KCS1, 2, 18 and 20, and showed in addition the capacity of KCS9 to produce C22 and C24 VLCFA [49,50]. Using such strategy, Blackblock and Jaworsky [50] were unable to unravel the activity of several KCS tested (KCS3, 4, 7, 16 and 19) and showed only very minor differences in the yeast fatty acid profile when expressing KCS11 and 17, whose chain length specificities remain unclear. 

The physiological characterization of arabidopsis *kcs* T-DNA mutant lines allowed not only for determining the chain length specificity of certain KCS but also the main plant lipid pools affected in the mutant plant. Often, the impacted lipid pool correlated well with the spatiotemporal expression of the studied KCS. In agreement with the seed specific expression of *KCS18/FAE1* and its heterologous expression in yeast leading to the accumulation of saturated and monounsaturated C20 and C22 VLCFA products, seed storage lipids from the *kcs18/fae1* mutant were nearly free of C20 and C22 VLCFA [34]. The *kcs6/cer6/cut1* mutant was shown to display one of the most severe waxless phenotype with stems completely devoid of wax crystals, a 90% decrease in stem wax content, and an accumulation of C24 and C26 derivatives at the expense of the longer typical wax compounds found in the wild type background [52]. This phenotype perfectly fits with KCS6 catalyzing in yeast the production of C26 and C28 VLCFA [47] and being highly expressed in epidermal tissues [53]. *AtKCS2* and *20* were shown to be highly expressed in root endodermis, and the *kcs2* and *kcs20* single mutants had reduced levels of C22 monomers in suberin [54,55], in line with the results obtained in in vitro and in vivo yeast assays [47,48,49]. Similarly, the *kcs1* mutant showed reduced levels of C20 and C22 VLCFA and derivatives in roots, while C16 and C18 fatty acids increased, as expected, from the results obtained through heterologous expression in yeast [47,48,56]. The *kcs1* mutant also displayed decreased levels of certain C26 to C30 wax compounds [56]. Nevertheless, this phenotype is most probably not directly due to the chain length specificity of AtKCS1 but rather to a lower substrate pool for KCS6 in the *kcs1* background. Finally, acyl-CoA profiling of *kcs9* roots showed a slight accumulation of 22:0-CoA species [57], in agreement with the KCS9 chain length specificity deduced from in vitro yeast assays [49]. The characterization of the various lipid pools containing VLCFA and derivatives in the *kcs9* mutant showed in addition that cuticular waxes, root suberin, sphingolipids and phospholipids compositions were all affected [57], suggesting that the VLCFA produced by KCS9 are subsequently been used in many lipid biosynthetic pathways. Very recently, AtKCS4 was shown to be involved in the elongation of VLCFA with more than 24 carbons and to be important for pollen tube and root growth [58]. Looking at the lipid phenotype of arabidopsis *kcs* mutants to unravel chain length specificity can nevertheless lead to conflicting results. Whereas Hegebarth and coworkers [51] reported that AtKCS16 could be involved in the production C36 and C38 carbons acyl precursors for leaf trichome and pavement surface wax, results from Lv and coworkers [59] suggest that AtKCS16 produces C22 and C24 VLCFA important to support lateral root development. It is nevertheless possible that other proteins, like the CER2-like proteins, allow AtKCS16 to produce both VLCFA chain lengths.

Altogether, the chain-length specificity was characterized for 2 AtKCSs by heterologous expression in yeast, for 2 others by lipid phenotyping of arabidopsis *kcs* mutants, and for 6 AtKCS by both approaches. Several important conclusions can in addition be drawn from the many studies on AtKCS conducted in yeast or in planta. First, they collectively gave clues about the catalytic activity of only 10 AtKCS, the products generated by the other 10 as well as their physiological roles in planta remaining completely unknown. Second, each KCS seemingly completes only a limited number of elongation cycles (2–3) so that the elongation of VLCFAs from C20 to C28 relies on different KCS with overlapping substrate specificities (Figure 2). Third, functional redundancy occurs between the different AtKCSs. Despite very strong homologies in their amino acid sequences, a phylogenic analysis sorted AtKCS proteins in eight subclasses [30], which may share some substrate specificity. For example, KCS2 and KCS20, both producing C20 to C24 VLCFA, group in clade ζ; KCS9, KCS4 and KCS17, producing C22 to C26 VLCFA, group in clade α; and KCS5 and KCS6, both producing C24 to C28 VLCFA, group in clade γ. Fourth, most AtKCSs are broadly expressed and involved in various lipid biosynthetic pathways. Indeed, only a very few KCS, such as KCS6, which are highly expressed in the epidermis of aerial organs, or KCS18, whose expression is seed-specific, seem to produce VLCFA for a restricted function. Overlapping substrate specificities and expression patterns lead to functional redundancy, which most probably prevents determining the exact biochemical activity and in planta function of most AtKCS.

### 1.4. Lipids Incorporating VLCFAs

**Figure 3:**
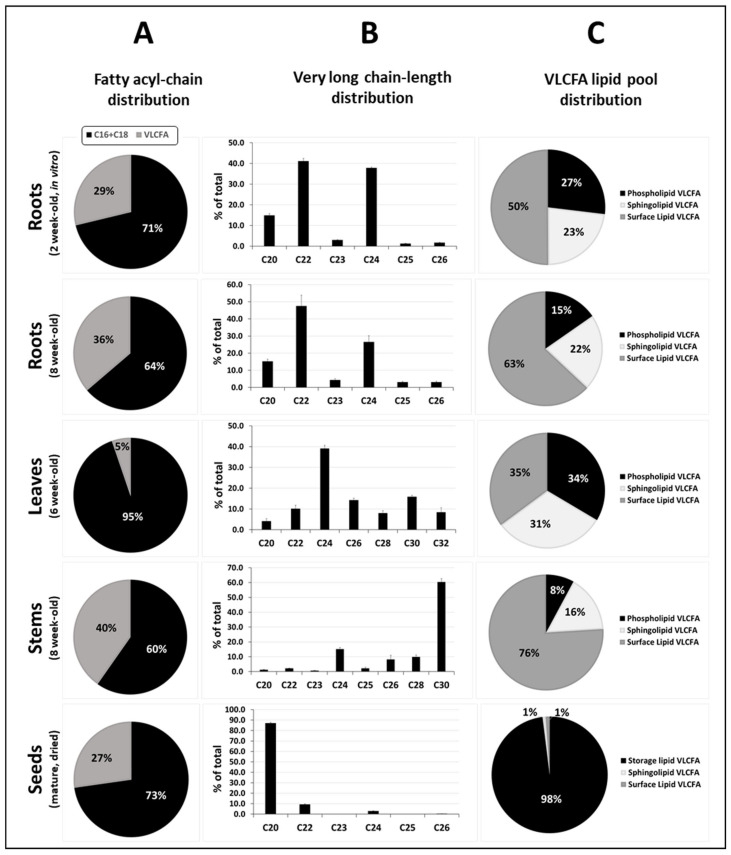
VLCFA content and distribution in *Arabidopsis* organs. (**A**) Distribution of long-chain (C16 and C18) fatty acids and VLCFA in *Arabidopsis* tissues (% of total); (**B**) VLCFA chain length distribution in *Arabidopsis* tissues (% of total); (**C**) Main lipid pools containing VLCFA in *Arabidopsis* tissues (% of total); Data for roots and stems were calculated from Delude et al. [60]. Note that for roots, the suberin polymer and soluble lipids were isolated and separately analyzed, while for stems waxes were first extracted, and waxes and dewaxed stem were separately quantified. For leaves and dried seeds global acyl-chain profiling, acyl-chain was released by transmethylation in 5% sulfuric acid in methanol for 3 h at 85 °C and silylated before GC analysis as for dewaxed stems in Delude et al. [59]. Unmodified C20 to C24 VLCFA were considered as phospho- or storage lipids, 2-hydroxy VLCFA as sphingolipids VLCFA, and typical waxes and suberin monomers as surface lipids VLCFA. For leaves and stems, the chain-length of the products from the decarbonylation pathway was considered as *n* + 1 (i.e., alkane C29 was counted in C30 VLCFA and derivatives).

To illustrate the importance and distribution of VLCFAs in plants, we quantified all fatty acid and fatty acid derivatives in different tissues of arabidopsis using GC-MS and -FID for identification and quantification, respectively. Data for roots and stems were calculated from a previous study [60], while new analyses were performed for leaves and dried seeds for this study. For global acyl-chain profiling, tissues were directly transmethylated in 5% sulfuric acid in methanol for 3 h at 85 °C, and the released acyl-chains were extracted and silylated before GC analysis. This procedure allows for an estimation of all the major acyl-chains present either in classical membrane phospholipids (as unmodified fatty acid), in the sphingolipids of the plasma membrane (as 2-hydroxy fatty acids) or in surface lipids (as waxes and cutin/suberin monomers). As shown in Figure 3A, VLCFA and derivatives accounted for 29 to 40% of all acyl-chains in most tissues, with the exception of leaves where their content was only 5% of the total due to the preponderance of thylakoid membranes rich in C16 and C18 polyunsaturated fatty acids in chloroplastidic lipids. The high proportions of VLCFA and derivatives in roots and stems is related to their very active metabolism in surface lipids (for suberin and cuticular wax biosynthesis, respectively), which resulted in surface lipids accounting for 50 to 63 and 76% of all VLCFA present in roots and stems, respectively (Figure 3C). Similarly, arabidopsis seeds containing high proportions of VLC monounsaturated fatty acids in storage TAGs, which represent by far the major lipid pool of seeds, 97% of the VLCFA present in seeds, were in storage lipids (Figure 3C). 

Arabidopsis organs also differed in terms of VLCFA and derivatives chain length (Figure 3C). In roots, the VLCFA and derivatives content as well as its proportion in surface lipids increased with root aging (Figure 3A,C), in agreement with the predominant role of suberin in root global lipid metabolism [60]. Therefore, C22, which is globally (i.e., unmodified and in the form of ω-hydroxy fatty acid, α,ω-dicarboxylic acid and fatty alcohol) [25] the most abundant VLC-length in arabidopsis roots suberin was also the major root VLC-length detected (Figure 3B). C20 and C24 also represented abundant VLC-lengths in roots. Such chain lengths are present in several lipid pools like membrane lipids (as unmodified chain), sphingolipids (as 2-hydroxy derivatives) and suberin. In leaves, sphingolipids and membrane lipids together accounted for about 65% of all the VLCFA and derivatives, while surface lipids contained 35% of the total (Figure 3C). In agreement, C24, which is the major VLCFA found in both membrane lipids and sphingolipids, was the most abundant VLC-length in leaves, representing about 40% of the total. In addition, significant amounts of VLCFA and derivatives with 26 to 32 carbons, corresponding to cuticular waxes, were detected (Figure 3B). In arabidopsis, stems are heavily covered with crystal waxes and generally contain about 16 times more epicuticular waxes than leaves, about 80% of these being 29 carbons-long and issued from the decarbonylation pathway [61]. Consequently, epicuticular waxes represented about 76% of all VLCFA and derivatives present in stems, and C30 was by far the most abundant chain-length (60% of the total). C24, either present in membrane phospholipids or sphingolipids, was the next most abundant chain length found in stems. Finally, in arabidopsis seeds, storage lipids in the form of TAG, which are much more abundant than all other lipid pools, were particularly rich in C20 and C22, which together accounted for about 95% of the total VLCFAs (Figure 3A,C). In addition, traces of suberin monomers (C22 and C24 ω-hydroxy fatty acid, α,ω-dicarboxylic acid) and of 2-hydroxy fatty acids from sphingolipids (C22 to C26) were also detected. Nevertheless, cuticular waxes present at the surface of the seeds could not be detected by this procedure because of their too low abundance.

Altogether, these data show that VLCFA and derivatives generally represent an important part of arabidopsis acyl-chain metabolism (Figure 3A), and that the different organs differ in terms of proportions as well as in the lipid pools containing these compounds (Figure 3C). The VLC-length distribution of the different organs (Figure 3B) reflects the major VLCFA and derivatives pools present in the organ under consideration (Figure 3C), but all organs contain substantial amounts of C22, C24 and C26-chain length due to the ubiquitous presence of phosphatidylserine and sphingolipids.

## 2. VLCFA-Derived Surface Lipids Constitute the Border between Plants and Its Surrounding Environment

### 2.1. Role of Cutin and Waxes in Aerial Plant Responses to Abiotic and Biotic Stresses

A major sink for VLCFA is the cuticle produced by epidermal cells and covering all the aerial organs of plants. Although the primary function of the cuticle is to limit non-stomatal water loss, its strategic positioning at the plant/air interface makes the cuticle a major player in plant/environment interactions such as responses to drought, high-dose visible light and UV radiation and pathogen or insect attacks [62]. The protective capacities of the cuticle are based on the physical and biochemical properties of its two highly hydrophobic components, cutin and cuticular waxes, which are finely assembled ultrastructurally in several layers [63]. The cutin polymer, with embedded intracuticular waxes, constitutes the cuticle proper and is connected to the cell wall by a cuticular layer made of cutin and polysaccharides. Covering the cutin matrix, the outermost layer of the cuticle is composed of epicuticular waxes that can form wax crystal microstructures. Cuticle synthesis starts in early stages of embryo development and is tightly co-regulated with plant growth to provide constant cuticle deposition [64]. Although the biochemical composition and the thickness of the cuticle vary among different plant species and/or among organs and developmental stages, a set of primary compounds is ubiquitously found in plant cuticles. Cutin, which provides the main mechanical strength of the cuticle, is a three-dimensional biopolyester of C16 and C18 polyhydroxy- and epoxyhydroxy-fatty acids cross-esterified to each other or via glycerol backbones, and usually poor in VLCFA and derivatives [65]. In contrast, cuticular waxes consist of a complex mixture of homologue series of VLCFA and derivatives, as well as non-acyl lipid cyclic components including terpenoids and flavonoids [16,17,66,67].

If VLCFA can be directly incorporated in the form of free fatty acids into cuticular waxes, they are mainly converted into a large number of derivatives such as alkanes, aldehydes, ketones, alcohols and esters before being incorporated into the cuticle. In other words, although VLCFA are precursors of all aliphatic wax components, their abundance in cuticular waxes is highly variable according to the plant species considered. For example, VLCFA account only for about 2 and 8% of all aliphatic compounds in arabidopsis stem and leaf waxes, respectively [61], but represent the most abundant acyl-chain class in Sorghum sheath (42.8% of the total, [68]). For cuticular wax biosynthesis, VLC-acyl-CoAs derived from fatty acid elongation are converted into several aliphatic derivatives through two distinct pathways: the alcohol-forming pathway and the alkane-forming pathway [16] (Figure 1 and Figure 2). The alcohol-forming pathway produces even-numbered fatty alcohols and wax esters, while the alkane-forming pathway produces aldehydes and odd-chain numbered alkanes, secondary alcohols and ketones. In arabidopsis, primary alcohols, with chain-length of 26 to 32 carbons, account for 12–14% of total waxes, whereas alkyls esters, with chain-length ranging from C38 to C48, are only minor components (<5%). In contrast, alkanes and derivatives, with chain length predominantly between 27 and 33 carbons, account for more than 80% of the total wax amounts. In arabidopsis, alkanes are major components representing up to 70% of the total waxes in rosette leaves [16]. Once produced in the endoplasmic reticulum, all these wax molecules are transported to the plasma membrane, secreted and cross the cell wall to form the outermost layer of epidermal cells.

Wax component chain-lengths vary from 24 to 38 carbons, suggesting that VLCFA precursors originate from various elongase complexes. Several KCS have been shown to be involved in wax synthesis in arabidopsis and crops (KCS1, KCS2, KCS6, KCS9, KCS10, KCS16 and KCS20) [7,17,51]. Additional genes, *KCS3, KCS5, KCS8, KCS12* and *KCS19*, showed high expression in young stem epidermal cells [13] that actively produce cuticular waxes but have not been functionally characterized. Based on expression patterns, mutant phenotypes and biochemical characterization, KCS6 appears to be the major KCS involved in the elongation of fatty acids longer than 26 carbons for the production of cuticular waxes. Indeed, a major reduction of KCS6 activity in *cer6* mutants or transgenic plants nearly abolishes wax accumulation in arabidopsis stems and results in conditional male sterility suggesting no functional overlap of KCS6 with other KCS [52,69]. However, heterologous expression of KCS6 in yeast was found to only produce fatty acids up to 28 carbons [45]. The characterization of the CER2-LIKEs gene family in arabidopsis, but also in rice, lotus, maize or poplar, has revealed that proteins encoded by these genes physically interact with the FAE components and play a key role in specifically modifying the chain-length specificity of KCS6, enabling the production of VLCFA longer than 28 carbons [40,42,70,71,72,73].

Consistent with the numerous roles played by the cuticle in plant/environment interactions, the amount of cuticular wax is highly regulated in response to environmental constraints (reviewed in [62,74]). Water deprivation, osmotic stress and ABA treatments have been shown to enhance wax accumulation through transcriptional regulation of arabidopsis wax metabolism [75]. Specifically, light and osmotic stresses have been shown to up-regulate *KCS6* expression [53], while several other *KCS* as well as *CER10* and *KCR1* transcripts have been shown to accumulate under NaCl, dehydration and mannitol treatments, and to decrease under low temperature and darkness conditions [30]. Since modification of *KCS* gene expression affects plant stress response, KCS not only play an important role in the synthesis of VLCFA precursors involved in wax synthesis but also in stress tolerance. For instance, arabidopsis *kcs1* mutants showed less resistance to low humidity conditions at a young age [56], while overexpression of *BnKCS1-1* or *BnKCS1-2* promotes wax production and increases drought tolerance in *rapeseed* by reducing water loss [76]. Transgenic peanut plants overexpressing *AhKCS1* showed increase in wax content and reduction in water loss compared to non-transgenic plants, suggesting that AhKCS1 plays a major role in peanut drought stress response [77]. Similarly, transgenic arabidopsis plants ectopically expressing a navel orange (*Citrus sinensis*) KCS6 (*CsKCS6*) displayed increased survival under drought and salt stress treatment [78], while barley *HvKCS6* mutants showed a slightly better growth rate in water limitation conditions [79]. Moreover, arabidopsis transgenic plants co-overexpressing *KCS1* and *AKR2A* exhibited a greater chilling tolerance than the plants overexpressing *AKR2A* or *KCS1* alone, as well as the wild type, whereas *akr2a kcs1* double mutants showed the poorest performance under chilling conditions [46]. These results indicate that *KCS1* and *AKR2A* are involved in chilling tolerance via modification of VLCFA biosynthesis. Finally, VLCFA-derived wax components were also shown to affect plant response to biotic stress since mutation in *HvKCS1* and *HvKCS6* in barley influenced the water barrier properties of the cuticle, which in turn affected the germination of barley powdery mildew fungus [80,81].

Despite the fact that the regulatory network controlling VLCFA biosynthesis is not fully known, several transcription factors involved in this regulation have been characterized. Data obtained from Kosma and co-workers [75] revealed a correlated up-regulation of cuticle-associated genes by water deprivation and ABA treatments, indicating that this phytohormone is likely to mediate the drought signal to wax biosynthesis. Characterization of the MYB96 transcription factor provided evidence for its function in ABA dependent regulation of wax synthesis during water stress in arabidopsis and *Camelina sativa* [20,82,83]. MYB96 has a strong impact on plant tolerance to abiotic stresses and regulates a variety of ABA responses, including lateral root development, stomatal movement, hormone biosynthesis and cuticular wax biosynthesis. Under drought conditions, MYB96 enhances the expression of several wax-associated genes and notably VLCFA elongase component encoding genes such as *KCS1, KCS2, KCS6* and *KCR1*, by directly binding to the consensus motifs of their promoters [20]. Another transcription factor that is increased by drought and ABA treatment, MYB94, has also been reported as a positive regulator associated with wax biosynthesis, its direct target genes being *KCS2, CER2* and *ECR* [84]. In maize, mutation of *ZmMYB94* altered cutin and wax biosynthesis at the coleoptile stage and resulted in fusion events between juvenile leaves [85]. Transcriptomic analysis of this mutant further showed that many genes involved in waxes and cutin biosynthesis and export were downregulated, including several genes from FAE complexes. This set of data collectively indicates that MYB94 and MYB96 exert an additive positive effect on cuticular wax biosynthesis, which represents an efficient adaptive mechanism of response to drought in plants [86]. Conversely, diurnally controlled DEWAX encoding an AP2/ERF-type transcription factor was reported to regulate wax biosynthesis negatively during daily dark/light cycles [87]. Then in the dark, DEWAX represses the expression of several wax-associated genes and VLCFA elongation related genes such as *KCS1, KCS2, KCS6, KCR1, ECR* and *CER2*. DEWAX was shown to directly bind to the *ECR* promoter by ChIP assay [87].

### 2.2. Role of Suberin in Plant Responses to Abiotic and Biotic Stresses

Another important lipid pool comprising considerable amounts of VLCFA and derivatives is suberin [14]. Suberin is another plant surface lipid barrier, which also comprises a biopolyester mainly made of oxygenated fatty acids and associated soluble waxes. Nevertheless, in contrast to the cuticle, suberin also contains a polyphenolic domain mainly composed of ferulic acid and is deposited close to the plasma membrane on the inner face of primary walls [15]. Suberin deposition occurs in root endodermal and periderm cell walls, in the seed coat protecting the embryo of seeds, in the bark of the cork oak (*Quercus suber*) tree and in specialized underground storage organs such as potato (*Solanum tuberosum*) tubers. Suberin deposition is also visible on the surface of fruits like apples and pears russeting varieties and in certain melons displaying suberized regions forming patterns of reticulation. Finally, suberin is produced as a healing polymer in response to mechanical stress and in abscission zones.

While absent in cutin polyesters, VLCFA and derivatives such as VLC-fatty alcohols, -ω-hydroxy acids and -dicarboxylic acids are considered to be indicative of suberin. In arabidopsis, C20 and C22 VLCFA and derivatives predominate in root suberin, while the suberin of the seed coat also contains large amounts of C24 VLCFA and derivatives. The exact same chain lengths of VLCFA and derivatives are enriched in reticulated melon and russeting apple skins when compared to smooth melon and waxy apple skins, respectively [88,89]. The situation is different in potato tuber and oak cork suberin, in which hydroxyl- and dicarboxylic acids are highly dominated by C18-species [90,91]. Nevertheless, potato tuber suberin also contains considerable amounts of C26 to C30 fatty acids and fatty alcohols in the polymer, as well as associated as soluble waxes in the form of free fatty acids, fatty alcohols and alkylhydroxy-ferulates [91]. In arabidopsis root suberin, C20 to C22 VLCFA and derivatives are present as components of the polyester as well as associated waxes in the form of alkyl-hydroxycinnamates [60].

Since VLCFA and derivatives represent about 40% of all suberin aliphatics, and fatty acid elongation represents an important step in the suberin biosynthesis pathway. Using promoter::GUS reporter lines, Franke and coworkers reported that at least 7 KCS (*KCS1, 2, 4, 8, 11, 20* and *21*) displayed strong expression in roots although 2 to 3 KCS should be enough to produce the “longest” (C24) arabidopsis suberin monomers [92]. Reverse genetic approaches based on the characterization of T-DNA lines confirmed that three of these seven candidates, *KCS1, KCS2* and *KCS20*, are involved in suberin biosynthesis. Using total FAMES analysis, Todd and coworkers [56] reported that the roots from the *kcs1-1* mutant were enriched in C16 and C18 fatty and dioic acids, whereas 22:0 VLCFA levels were decreased. Using a similar analytical approach, Shang and coworkers [93] reported 30 to 60% decrease in the levels of 18:0, 20:0, 22:0 and 24:0 in the *kcs1-2* mutant. More recently, Trinh and coworkers [4] described precisely the impact of *KCS1* mutation on the global root acyl-chain composition and reported that the amounts of C16 chain length fatty acids and derivatives increased, while those of VLCFA decreased, suggesting a role for KCS1 in the elongation of C16 to C18 in roots.

Studies aiming at characterizing candidate genes responsible for suberin biosynthesis unambiguously showed that KCS2 and KCS20 are both involved in generating suberin precursors and monomers. Roots from *kcs2* and *kcs20* mutant lines showed reduced growth and their suberin composition was characterized by decreases in C22 and C24 VLCFA and derivatives, while the amounts of monomers with 18 and 20 carbon atoms increased [54,55]. The effects were apparently additive in the *kcs2 kcs20* double mutant, but the fact that its suberin still contained relatively high levels of VLCFA and derivatives suggests that additional KCS are involved in suberin biosynthesis or activated for maintaining suberin levels close to wild-type in these mutant backgrounds. It should also be reported that Franke and coworkers [54] showed that the expression of KCS2 was up-regulated by osmotic, drought and wounding treatments, in agreement with suberin being deposited in response to various abiotic and biotic stresses. Finally, Lee and coworkers [55] reported that the *kcs2* and *kcs20* as well as the double *kcs2 kcs20* mutants were also affected in cuticular waxes, suggesting that both genes are not just involved in suberin metabolism but also important in generating the above ground cuticular barrier. Similarly, Kim and coworkers [57] reported that *KCS9* was expressed in various organs and tissues and involved in the synthesis of C24 VLCFA, which are present in most plant lipid pools, so that its mutation affected the composition of suberin, cuticular waxes, sphingolipids and phospholipids. The effect of *kcs9* mutation on these different lipids was nevertheless minor since it slightly affected the chain-length distribution of all these pools but not their total amounts. In agreement, the level of *KCS9* transcripts was not significantly altered following applications of osmotic, salt, and drought stresses [57], suggesting that KCS9, in contrast to KCS2 and 20, may not be a major player in the plant response to abiotic stress.

Suberin is also known to be deposited to seal novel aboveground surfaces appearing naturally during plant development, for example, in abscission zones, or accidentally because of mechanical wounding. This wound-healing mechanism primarily serves as a protection against pathogen infection to sustain post-injury survival. Wound-healing potato tuber discs have indeed been used for decades as a laboratory model for studying suberin biosynthesis and regulation (reviewed in [94]). Schreiber and coworkers [95] showed with wounded potato tuber discs that periderm formation was accompanied by clear increases in the total amounts of soluble waxes and suberin monomers as well as in the average chain length of their linear aliphatics. In particular, the authors reported that the amount of 30-carbons alkyl-ferulates tripled three weeks after wounding. More recently, it was shown that the *StKCS6* gene was induced 2 days post-wounding, implying that VLCFA biosynthesis is up-regulated in response to mechanical damages. In the same line, a study of tubers from potato plants in which *StKCS6* had been silenced by RNA interference showed clear reductions in VLCFA and derivatives with 28 or more carbon atoms (≥C28) present in the suberin polymer and peridermal waxes, while shorter compounds increased [96]. Since the peridermal transpiration of these potatoes increased, these results show that StKCS6 is involved in the synthesis of monomers with 28 carbon atoms or more and that these VLCFAs and derivatives are important for suberin to function as hydrophobic barrier. Suberin is important for regulating water and nutrients absorption in roots, and several studies reported that suberization is induced under drought or waterlogging conditions [97,98,99]. Nevertheless, suberin is not only produced by plants in response to abiotic stress since ectopic suberization in the cell layers surrounding infection and feeding sites of cyst and root-knot nematodes has been reported [100]. The same study showed that the transcript abundances of *KCS2* and *KCS20*, as well as that of other well-known suberin genes (*CYP86A1, CYP86B1, FAR4, FAR5* and *GPAT5*), were increased in root segments containing infection sites, implying that suberin could play a role in defense against soil-borne pathogens.

## 3. VLCFA and Sphingolipids in Plant Responses to Abiotic and Biotic Stresses

### 3.1. VLCFA in Plant Sphingolipids

Plant sphingolipids form a significant portion of the lipids present in higher plants and constitute up to 40% of those making the PM [101]. They are basically characterized by a ceramide formed by a sphingoid base amide-linked to an acyl chain, which in arabidopsis is often a VLCFA hydroxylated in position 2 (Figure 1) [11,12]. In plants, sphingolipids can be divided into 4 major classes: free Long-Chain Bases (LCBs), CERamides (Cers), GLuCosylCERamides (GlcCers) and Glucosyl InositolPhosphoCeramides (GIPCs), representing ca. 1%, 6% 36% and 57%, respectively, of the sphingolipidome of arabidopsis seedlings [9,102,103]. Plant sphingolipids constitute a highly complex lipid class since they are composed of up to 9 different LCBs, 32 different fatty acids and various polar heads, which still need to be completely described in terms of exact sugar composition, kind, and stereochemistry of bounds. If several LC-MS methods based on multiple reaction monitoring mass spectrometry scan have been described to analyze the full sphingolipidome of plants [9,103,104,105,106,107], only relative quantification can be performed, and suitable standards for plant GIPCs are still lacking so adequate methods still need to be developed.

The first step in plant sphingolipids synthesis is the formation of the long-chain base (LCB) by the condensation of a serine with palmitoyl-CoA by the serine palmitoyl transferase (SPT) in the endoplasmic reticulum. The product of this reaction, 3-ketosphinganine, is then reduced to sphinganine (d18:0) by the action of the 3-ketosphinganine reductase [102]. The resulting LCBs display two hydroxyl groups at the C-1 and C-3 position and are referred to as dihydroxy LCBs (d18:0). A third hydroxyl group may be introduced at the C-4 position through the action of an LCB C-4 hydroxylase (producing a trihydroxy LCBs, t18:0), while double bonds may be introduced at the Δ4 and/or Δ8 positions by the activity of distinct LCB desaturases. In certain yeasts and algae, VLCFA-CoA can be used to produce very-long-chain base [108,109]. The second step in plant sphingolipids synthesis is catalyzed by the ceramide synthases that link LCB to a fatty acid via an amide bond. In arabidopsis, there are three ceramide synthases called LOH for *LOngevity Homologs* with different chain-length specificities. While LOH2 preferentially uses 16:0 as substrate, LOH1 and LOH3 are specific to VLCFA, and since they activity predominate, VLCFA represent nearly 80% of the fatty acids founds in sphingolipids, most of the rest being 16:0 [110]. The third step in plant sphingolipids consists in the addition of polar head substitutions on the first carbon of the ceramide to form complex glycosphingolipids. The glucosylceramide synthase (GCS) transfers a glucose producing the simple GlcCers [111], while for the complex GIPCs, the ceramide head group consists of a phosphate bound to an inositol, forming the inositol phosphoryl ceramide (IPC) backbone, with additional saccharides depending on the botanical classes [112]. In a general manner, monocot GIPCs contain 3 sugars, whereas dicot GIPCs contains 2 sugars but exceptions have been described [112]. Since GIPC-synthesizing enzymes are all located in the Golgi apparatus and the polar head is grafted within the lumen of this organelle, GIPC’s polar head faces the outer surface of the plant cell after secretion and fusion with the PM. Recent work on purified GIPCs showed by measuring their ζ-potential that they contribute significantly to the negative charge of the plant PM outer leaflet [111], and in this way might influence its interaction with cell wall components [113].

In arabidopsis, sphingolipids VLCFA are dominated by C24 species, 24:0 and 24:1, collectively representing nearly 80% of the total [111]. In addition, C22 and C26 species, representing each about 10% of the total, as well as minor amounts of odd-chain fatty acids (mainly C23 and C25) are often reported. Such chain–chain lengths are in agreement with the study of Kim and coworkers [57], which showed that KCS9 was involved in sphingolipid metabolism in arabidopsis. As stated above, about 80% of these VLCFA are hydroxylated in C2 position (α-hydroxylated; [11]). If most non-hydroxylated VLCFA are found in Cers, the most abundant arabidopsis sphingolipids (GlcCers and GIPCs) contain nearly exclusively α-hydroxylated VLCFA [103]. This particularity, easily detectable by GC-MS after silylation, facilitated sphingolipids purification and analysis in model plants [100]. The presence of VLCFA, 2-hydroxylated or not seems universal in all plant GIPCs analyzed [11,107]. In arabidopsis, two fatty acid 2-hydroxylases (FAH) have been described: AtFAH1, which preferentially adds a hydroxyl group to a VLCFA, and AtFAH2, which prefers 16:0 [114]. Both proteins reside in the ER and most probably use Cers as substrates. Another modification often observed in plant sphingolipids VLCFA is the presence of a ω9-desaturation, and the acyl-CoA desaturase ADS2 was shown to be responsible for this modification [115]. The pivotal role played by the VLCFA present in sphingolipids is easily highlighted by the fact that the *loh1-2 loh3-2* double mutant, which sphingolipids are nearly devoid of VLCFA, is not viable [103]. In contrast, the relevance of α-hydroxylation remains open as the *fah1 fah2* double mutant, which still contains minor amounts of α-hydroxylated fatty acids in sphingolipids due to a residual expression of FAH1, is affected in growth but remains viable [116].

### 3.2. Role of VLCFA-Sphingolipids in Structuring the Membranes

PM-resident signaling processes are spatially organized, and receptors, co-receptors and transducers are particularly enriched in nanodomains involved in the transduction of biotic and abiotic stimuli [117,118]. Together with sterols, sphingolipids play a key role in nanodomain formation by forming liquid ordered domains in the plasma membrane [119]. In plants, VLCFA-containing GIPC were hypothesized to strongly influence sterol-dependent nanodomain formation. Indeed, GIPCs were shown to be strongly enriched in detergent-insoluble membranes, the biochemical counterpart of PM nanodomains, in several plant models [12,101,120].

As GIPC are not commercially available, we recently developed a protocol based on previously published methods [11,121,122] to extract plant GIPC fractions from different plant species. LC-MS^2^ analysis confirmed the presence of trihydroxylated long-chain bases and α-hydroxylated-VLCFA with up to 26 carbons. Obtaining purified GIPCs opened the possibility to study the biophysical features of these intriguing lipids and to characterize their role in membrane models mimicking the outer leaflet of the plant PM. First, cryo EM and neutron reflectivity showed that in symmetrical liposomes the presence of GIPC significantly increase the thickness of the model membrane, likely because of their content in VLCFA. However, it should not be forgotten that native PMs from plants are asymmetrical as GIPC and sterol are only enriched in the outer leaflet, whereas GluCER and phospholipids reside in the inner one [101,123]. Nevertheless, it is highly likely that outer leaflet GIPC allows for the establishment of interdigitation (i.e., penetration of the methyl end of VLCFA into the inner leaflet [124]), as it has been described for gangliosides in animal membranes [125,126]. The significance of such interdigitation/pinning phenomena between the two leaflets is still under investigation in plants but may have importance consequences. Second, Langmuir monolayer and modeling studies showed differences between the spatial organization of the GIPC with free and conjugated phytosterols: the α-side of the steryl moieties of β-sitosterol was directed towards the LCB/VLCFA of the GIPC, whereas the steryl rings of stigmasterol was positioned at a perpendicular angle with respect to GIPC hydrocarbon chains [106]. This result is in good agreement with the propensity of sitosterol, but not stigmasterol, to promote liquid ordered domain formation [127,128].

### 3.3. Role of Sphingolipids in Abiotic Stress Responses

Although phytosterols were proposed a while ago to be essential for regulating the fluidity of plant plasma membrane in response to changing temperatures [129], solid state 2H-NMR recently showed that GIPC have also a high propensity to regulate the fluidity of the PM when temperature varies [107]. In fact, subtle changes in sphingolipids can induce radical effects on the development of arabidopsis when exposed to abiotic stress such as cold. For example, it was very recently demonstrated that the VLCFAs content in sphingolipids, precisely that of the monounsaturated C24 and C26 fatty acids, was increased in response to cold, with the enzyme catalyzing the desaturation reaction, AtADS2, being upregulated [130]. Similarly, LCB desaturation, in particular at the Δ8 position of the LCB by sphingolipid Δ8 LCB desaturases, was shown to play a role in cold tolerance in tomato [131] and arabidopsis [132]. Sphingolipid desaturation is therefore a way for plants to moderate the impact of freezing temperatures on the cell-delimitating membrane. On the contrary, a function for sphingolipids in plant tolerance to high temperatures has so far not been characterized, in contrast with the trienoic fatty acids present in the thylakoid membranes that have been shown to be involved in both chilling and high-temperature tolerance [133,134,135].

Another set of data revealed the unexpected role of GIPCs in response to salt stress. Salt stress in plants triggers an increase in cytosolic calcium concentration, which activates calcium-binding proteins and upregulates the Na^+^/H^+^ antiporter to remove the sodium ion from the inside of cells. By searching for arabidopsis mutants impaired in salt-induced calcium responses, the groups of Z. Hu and Z.-M. Pei isolated the mutant monocation-induced [Calcium]^in^ increases 1 (MOCA1) [136]. MOCA1 turned out to be a glucuronosyltransferase responsible for GIPC biosynthesis. The authors proposed that sodium ions bind to GIPCs to activate calcium channels. This mechanism might imply that GIPCs are involved in the plant adaption to various environmental salt levels and could be used to improve salt resistance in crops. Another hypothesis could be that sodium binding to GIPCs drives the formation of nanodomains that would alter the dynamics of signaling proteins involved in salt response, such as the activities of NADPH oxidases or GTPases [137]. The exact role of VLCFA grafted to GIPCs in this mechanism nevertheless remains to be determined.

Finally, several reports described GIPCs containing VLCFAs as essential for activation of hypoxia response and for protecting arabidopsis from hypoxic stress [138]. Other studies demonstrated that VLCFA unsaturation in ceramides is a defense strategy for hypoxic tolerance in arabidopsis [139,140].

### 3.4. Role of Sphingolipids in Biotic Stress Responses

A huge amount of genetic and biochemical data showed the importance of sphingolipids in biotic response and plant defense against fungi and bacteria pathogens by activating programmed cell death leading to spontaneous necrosis and lesions, also called accelerated cell death phenotype [141,142,143,144,145]. In particular, the MYB30 transcription factor was characterized as a positive regulator of the programmed cell death associated with hypersensitive response (HR) in arabidopsis [146] and shown to induce the expression of genes coding for all four core proteins of FAE complex and at least 3 KCS (*KCS1, KCS2* and *KCS10* and *KCR, PAS2* and *ECR*) within one hour following inoculation with an avirulent strain of Xanthomonas [147]. MYB30 therefore seems to activate HR-related cell death via VLCFA or derivatives, and sphingolipids, which represent an important VLCFA pool in leaves, were suggested to be involved as signaling molecules. The review of De Bigault Du Granrut and Cacas [148] proposed different scenarii by which sphingolipids could act in stress signaling, but the exact molecular mechanism still remains uncharacterized.

In animals, toxins killing the cells by forming pores in the PM were shown to use sphingolipids as receptors [149]. For example, toxins of sea anemones target animal sphingomyelin (absent in plants). Recently, a similar mechanism has been evidenced in plants for the plant deadly toxins from the Necrosis and ethylene-inducing peptide1–Like Proteins (NLP) family, which constitute one of the largest family of toxins secreted by oomycetes, bacteria and fungi. These toxins are well known to induce necrosis and ethylene production in dicot plants but not in monocot plants [150]. Biochemical analyses recently revealed that NLP proteins bind with high specificity to the extracellular sugar head groups of GIPCs [144]. Three-D protein analyses further showed that NLP binding to GIPC’s sugar head led to a protein structural change with the opening of a crevice between loop L2 and L3, close to where magnesium ion binds, allowing NLPs to bind to the plasma membranes. Interestingly, NLP can also bind to GIPC of monocots (with three sugars), but the longer sugar head groups preclude the NLP from interacting with the plasma membrane, leading to a lack of toxicity [151]. The role VLCFA in this interaction is not known, but the use of metazachlor inhibitor of KCS would be of great interest to study the role of fatty acid length in NLP toxicity.

## 4. VLCFA in Phosphatidylserine and in Plant Development

### 4.1. Phosphatidylserine Is Enriched in VLCFA

VLCFA can be also be found esterified in membranous phospholipids, in particular PS, where they account for 49 to 72% of all acyl-chains depending on the arabidopsis tissue considered [1]. Although PS represents less than 2% of polar glycerolipids in all tissues [1], this specific accumulation of VLCFA may have important biological functions. VLCFA have also been found in the much more abundant PE and PC, but with much lower abundance (5 to 8% and 1 to 11%, respectively [1]). Although the role of these phospholipids has been studied in many plant responses to abiotic and biotic stresses, whether the VLCFA they contain are of any importance in these processes has rarely been addressed.

If the exact significance of the presence of VLCFA in PS still needs to be addressed, Li and coworkers [152] showed that the acyl-chains of PS lengthened during development, being the longest before senescence, and following heat or dehydration stress. Similarly, in rice, the *es5* mutant displayed higher levels of PS and an early senescing phenotype, suggesting that PS could play a role in plant cell death signaling pathways [153]. PS is not ubiquitously distributed in plant cells and primarily found at the PM, where together with phosphoinositides, PS contributes to the electronegativity of the inner leaflet of the plant PM, which is the most electronegative compartment of the cell. This electrical feature is critical for the transient yet specific recruitment of proteins with polybasic regions, as recently exemplified for the small G protein Rho of Plants 6 (ROP6; [154]). High-resolution single-molecule imaging showed that ROP6 is stabilized by PS into PM nanodomains required for auxin signaling. PS content varies during plant root development and modulates the quantity of ROP6 nanoclusters induced by auxin, and hence downstream signaling, including regulation of endocytosis and gravitropism. This study therefore showed that PS acts as a lipid rheostat that fine-tunes Rho GTPase signaling in arabidopsis [154].

VLCFA-containing PS could also be important for generating interactions between the two leaflets of membranes, potentially through so-called interdigitation, although the interleaflet coupling remains to be fully understood. In animal cells, many studies have showed the cholesterol-dependent interactions between the VLCFA containing-sphingolipids in the outer leaflet and phosphatidylserine 18:0/18:1-PS in the inner leaflet [155]. The cross-linking of sphingolipids may result in clustering of PS, allowing for the transfer of signals from outside the cell into the cytosol. The exact role of VLCFA in PS in plants nevertheless remains to be determined.

### 4.2. VLCFA and Plant Developmental Processes

VLCFA have also been shown to affect several important developmental processes, which in some cases are initiated by the plant in responses to environmental changes or abiotic stimuli. Nevertheless, in most cases, the exact molecular mechanisms regulating these responses still need to be elucidated.

Plants are known to lower they stomatal density in response to elevated carbon dioxide atmospheric concentrations. Gray and co-workers [156] characterized an arabidopsis mutant that displayed a 42% increase in leaf stomatal density in response to a doubling of carbon dioxide concentration and named it *hic* for *high carbon dioxide*. The authors found that the gene responsible for the *hic* phenotype corresponds to KCS13, suggesting that VLCFA could be involved in this adaptive response. The current hypothesis is that KCS13/HIC would produce a specific VLCFA derivative inhibitor that would (or a specific VLCFA derivative that would allow for an inhibitor to) diffuse through the cuticle to preclude the stomatal cell fate of surrounding satellite meristemoids [157]. The specific roles of VLCFA in epidermal cells development are nevertheless difficult to assess since it seems impossible to separate epidermal cell fate specification from cuticle biogenesis, as exemplified by that the observation that several arabidopsis mutants affected in their cuticle also display epidermal defects [64]. Another example is the *fiddlehead* mutant, which is characterized by numerous fusion events between leaves and/or reproductive organs as well as twice less trichomes on leaves [158]. The *FIDDLEHEAD* gene codes for KCS10, and its products would in this case positively regulate the evolution of protoderm cells into trichomes, whose correct development would be important for preventing fusion events between epidermal tissues of organs tightly appressed to each other’s when arising from meristems. Unfortunately, the products and the exact biochemical activity of FIDDLEHEAD/KCS10 and HIC/KCS13 remain unknown so that the involvement of VLCFA or derivatives in these processes still requires to be demonstrated.

Another fundamental process where VLCFA and derivatives are of primary importance is fertilization, which is absolutely required for the sexual reproduction of higher plants. Once pollen grains reach the stigma of flowers, they need to hydrate and germinate before they penetrate through the papilla cells of the stigma to reach the ovule. The initial steps of this process can be seen as a fusion event between the intact cuticles of the pollen and stigma, and several arabidopsis mutants affected in their cuticle (*cer1*, *cer3* and *cer6*) were shown to be male-sterile under low-humidity conditions because their pollen grains would not become hydrated on stigmas [159]. A recent study showed that the expression *KCS6* and *CER2-like* genes in the endothelium of anthers is necessary for the production of ≥C26 VLCFA and derivatives, which accumulate on the surface on mature pollen where they can serve as signaling molecules to activate the transfer of water from the papilla cells of the sigma for pollen hydration.

The importance of VLCFA for root development was also demonstrated. First, Shang and coworkers [93] reported that VLCFA produced by KCS1, or derivatives, acts as a pericycle-specific signal to restrict the capacity of this tissue to form a callus, and consequently new organs by regeneration. The transcription factor PUCHI was then shown to control the expression of several genes involved in VLCFA biosynthesis, among which KCS1, and thus to regulate callus as well as lateral root development [4]. Lateral root emergence is a very complex developmental process initiated by pericycle founder cells and tightly controlled by auxin, auxin-responsive factors and AP2-ERF transcription factors, including PUCHI and ERF13. The recent study by Lv and coworkers [59] showed that phosphorylation of ERF13 eliminates its negative regulation on KCS16 expression, thereby promoting the biosynthesis of C24-VLCFA that seemingly supports lateral root emergence. Interestingly, both the *puchi-1* and *kcs1-5* mutants showed reduced levels of C24 2-hydroxylated fatty acids levels in their roots, suggesting that sphingolipids could be involved in the control of this program. In agreement with such a function, it has also been demonstrated that VLCFA-containing ceramides were mediating positional signals for correct protoderm/epidermis differentiation during lateral root emergence [160]. All these studies therefore support the idea that VLCFA or derivatives are important signals regulating different steps and tissue zones involved in the correct development of lateral root primordia.

VLCFA or derivatives may indeed not only control developmental programs but also plant responses to environmental signals, for example, the capacity of roots to orient their growth according to the gravity vector. Beside the PM, sphingolipids with α-hydroxylated VLCFA are enriched in certain secretory vesicles of the trans-Golgi network, a central hub organelle, divided into subdomains hosting distinct trafficking pathways (including polar delivery to apical membrane). By using the drug metazachlor that inhibits KCS activity (and therefore reduces the length of VLCFA), Wattelet-Boyer and coworkers [161] showed using transmission electron microscopy that the chain length of sphingolipid VLCFA influences the morphology and interconnections of TGN-associated secretory vesicles in arabidopsis roots. Sphingolipids were in addition shown to be critical for de novo polar secretory sorting of the auxin carrier PIN2 to the apical membrane of cells. Hence, sphingolipids links VLCFA in lipids of TGN subdomains via the polar secretory trafficking of PIN2 toward the apical membrane of polarized epithelial cells to the response plants to gravitropism [161].

## 5. Concluding Remarks

In this review, we showed that VLCFA and derivatives are important components of various sorts of lipids involved in many plant responses to biotic and abiotic stresses. Surface lipids such as suberin in roots or cuticular waxes in aerial parts contain large amounts of VLCFA and derivatives, whose biosynthesis is upregulated upon osmotic stress, playing a role in non-stomatal water loss control. Sphingolipids present in the PM outer leaflet are enriched in VLCFA and form nanodomains playing a primary role in intercellular communication and signal transduction. Although PS represents less than 2% of polar glycerolipids in all tissues [1], the fact that it specifically accumulates VLCFA may have important biological consequences.

Although biochemical studies clearly showed that the KCS substrate specificity dictates the chain length of the VLCFA produced, the number of *KCS* genes present in plant genomes usually largely overpasses the minimum number of KCS needed to produce all the chain lengths found in VLCFA and derivatives. Studies conducted in arabidopsis clearly showed high functional redundancy as well as broad spatiotemporal expressions for most KCS: more than half of arabidopsis KCS isoforms are ubiquitously or almost ubiquitously expressed throughout the plant with variable intensities in the different tissues, while the remaining ones show expression profiles restricted to reproductive organs (flower and siliques; [30]). This implies a very complex regulation of arabidopsis *KCS* gene expression in the different tissues and along plant development, and may explain why most arabidopsis *kcs* mutants do not display very strong phenotypes or any. In addition, since C20 to C26 VLCFA are being incorporated in different lipid pools (phospholipids, sphingolipids and surface lipids), alterations of VLCFA levels have pleiotropic consequences in plants, which complicates the study of the role of specific VLCFA or VLCFA-producing complexes.

The various functions played by VLCFA-containing lipids are just emerging, and most remain to be discovered. The interconnection between VLCFA-surface lipids and epidermal differentiation deserves further attention, and acyl-CoA profiling or VLCFA-targeted lipidomic tools could provide important hints regarding the specific roles of epidermal VLCFA in plant responses to environmental changes. Biophysical and modelling approaches could help to unravel how VLCFA present in sphingolipids (outer leaflet) and phosphatidylserine (inner leaflet) structure PM organization and participate in transmembrane signal transduction. Finally, the CRISPR-Cas9 technology could help address the exact function of each KCS in VLCFA biosynthesis through the generation of multiple mutant or cell-specific deregulated lines.

## Data Availability

Not applicable.
